# Making sense of frailty: An ethnographic study of the experience of older people living with complex health problems

**DOI:** 10.1111/opn.12172

**Published:** 2017-10-09

**Authors:** Julie Kathryn Skilbeck, Antony Arthur, Jane Seymour

**Affiliations:** ^1^ Sheffield Hallam University Sheffield UK; ^2^ University of East Anglia Norwich UK; ^3^ University of Sheffield Sheffield UK

**Keywords:** frailty, long‐term conditions, nurse, nursing, older people, resilience, transitions

## Abstract

**Aim:**

To explore how older people with complex health problems experience frailty in their daily lives.

**Background:**

A better understanding of the personal experience of frailty in the context of fluctuating ill‐health has the potential to contribute to the development of personalised approaches to care planning and delivery.

**Design:**

An ethnographic study of older people, living at home, receiving support from a community matron service in a large city in the North of England.

**Methods:**

Up to six care encounters with each of ten older people, and their community matron, were observed at monthly intervals, over a period of time ranging from 4 to 11 months. Semi‐structured interviews were conducted with the older participants in their own homes. Fieldwork took place over a 4‐year period. Data analysis was undertaken using the constant comparative method.

**Findings:**

The experience of frailty was understood through the construction of four themes: *Fluctuating ill‐health and the disruption of daily living; Changes to the management of daily living; Frailty as fear, anxiety and uncertainty; Making sense of changes to health and daily living*.

**Conclusions:**

Older people work hard to shape and maintain daily routines in the context of complicated and enduring transitions in health and illness. However, they experience episodic moments of frailty, often articulated as uncertainty, where daily living becomes precarious and their resilience is threatened. Developing an understanding of the personal experiences of frail older people in the context of transition has the potential to inform nursing practice in person‐centred care .

**Implications for practice:**

Nurses need to support frail older people to maintain independence and continuity of personhood in the context of daily routines.

**What does this research add to existing knowledge in gerontology?**

- Frail older people work hard to shape and maintain daily routines in the context of complicated and enduring transitions in health and illness.
- Frail older people experience episodic moments of frailty in their daily lives where daily living becomes precarious.

**What are the implications of this new knowledge for nursing care with older people?**

- When planning personalised care for frail older people their experience of fluctuating ill‐health must be explored.
- Nurses must look for opportunities to enhance the coping strategies of frail older people by focusing on situations that challenge their resilience.

**How could the findings be used to influence policy or practice or research or education?**

- Policymakers need to consider how frail older people manage their experience of fluctuating health and enduring transitions.
- Further longitudinal research, rather than a cross‐sectional approach, is required to explore how nurses can utilise the personal experience of frailty in assessment and care planning.
- The findings highlight the knowledge, skills and attitudes that nurses require to care for frail older people.

## 1 INTRODUCTION

Given the global importance of developing an understanding of how best to organise and deliver health and social care to frail older people the dearth of literature exploring the experience of frailty from an older person's perspective is surprising. Definitions of frailty remain a subject of debate, although its multifactorial nature, with physical, social, psychological and spiritual dimensions, has been widely documented (Browne & Markle‐Reid, 2003; Levers, Estabrooks, & Ross Kerr, 2006). Nevertheless, within clinical practice, there is a tendency to view frailty within a narrow biomedical and functional framework.

Frailty is recognised as a state of increased vulnerability, comorbidity and poor health outcomes (Clegg, Young, & Lliffe, 2013) with important implications for the delivery of appropriate health and social care services (Cornwell, 2012). Frailty typically occurs in the “fourth age,” a period that has been reported in the literature as characterised by senility, a loss of agency and associated with transitions to impairment, ill‐health, decline and advanced ageing (Laslett, 1989; Gilleard & Higgs, 2010; Higgs and Gilleard 2015). Grenier (2012) has reported that it is compounded further by situational constraints, including poverty, social isolation and increased dependency. This paper contributes to an understanding of the personal experience of frailty in the context of fluctuating ill‐health.

## 2 BACKGROUND

Decline in health and well‐being, including physical, psychological and social functioning, has been identified as a key component of the personal experience of frailty in later life (Becker, 1994; Puts et al., 2009; Warmoth et al., 2016). Frail older people articulate a sense of loss associated with the decline in overall health and well‐being (Nicholson, Meyer, Flatley, & Holman, 2012). Taube, Jakobsson, Midlov, and Kristensson (2016) highlight that frail older people experience loneliness as a consequence of increasing physical impairment. The experience of decline is punctuated by transitions in health and illness (Grenier, 2012) yet the experience of disruption to health is diverse and typically dependent on the onset, nature and pattern of the transition trajectory (Godfrey & Townsend, 2008; Nilsson, Sarvimaki, & Ekman, 2000). Lunney, Lynn, and Foley (2007) and Thomas et al. (2010) identified how a particular “frailty” trajectory is a common pattern and route to death in later life.

Several recent papers have identified how maintaining a balance between autonomy and dependence on others for care when frail is complex. Results of an interview study by Nicholson et al. (2012) showed how older people living at home used a variety of strategies to create anchorage in their daily lives. Ebrahimi, Wilhelmson, Eklund, Moore, and Jakobssen (2013) have reported how older people's bodies become unpredictable and how this influences the ability to manage daily living. Furthermore, frail older people often have little control of the decline in their health and well‐being (Puts et al., 2009) and a period of hospitalisation often threatens their ability to engage in daily routines and activities (Andreassen, Lund, Aadahl, & Sorensen, 2015; Nicholson, Morrow, Hicks, & Fitzpatrick, 2017). It can be challenging for frail older people to make sense of their lives in the context of ill‐health; examining this among older women, Grenier (2006) concluded that they made sense of their life events in relation to their diverse experiences and identities, rather than as a frail person. This ethnographic study, drawing on work undertaken for a doctorate (Skilbeck, 2014), explored how older people with complex health problems experienced frailty in their daily lives.

### 2.1 Design

Ethnography was used for a focused and intensive analysis of how frail older people experience and attribute meaning to their daily life. A central tenet of ethnography is that individuals’ experiences are socially organised (Hardin & Clarke, 2012). The researcher (JKS) began by examining those experiences, focusing data collection on individuals, and simultaneously examined the context and the role of others in shaping the experience (Burns & Groves, 2009); exploring how broader social relations have shaped them (Brewer, 2000; Hammersley & Atkinson, 2007).

### 2.2 Setting and participants

Access to home‐dwelling frail older people was gained through a community matron team in a city in the North of England. This city has a population of 555,500; at the time of the study, there were approximately 40,122 people aged over 75. Of these, 7,206 were aged between 85 and 89 years and 3,433 were aged over 90 (ONS 2010). In the UK, community matrons are nurses who provide one of the main services to older adults with complex problems at home (Department of Health 2005).

**Figure 1:**
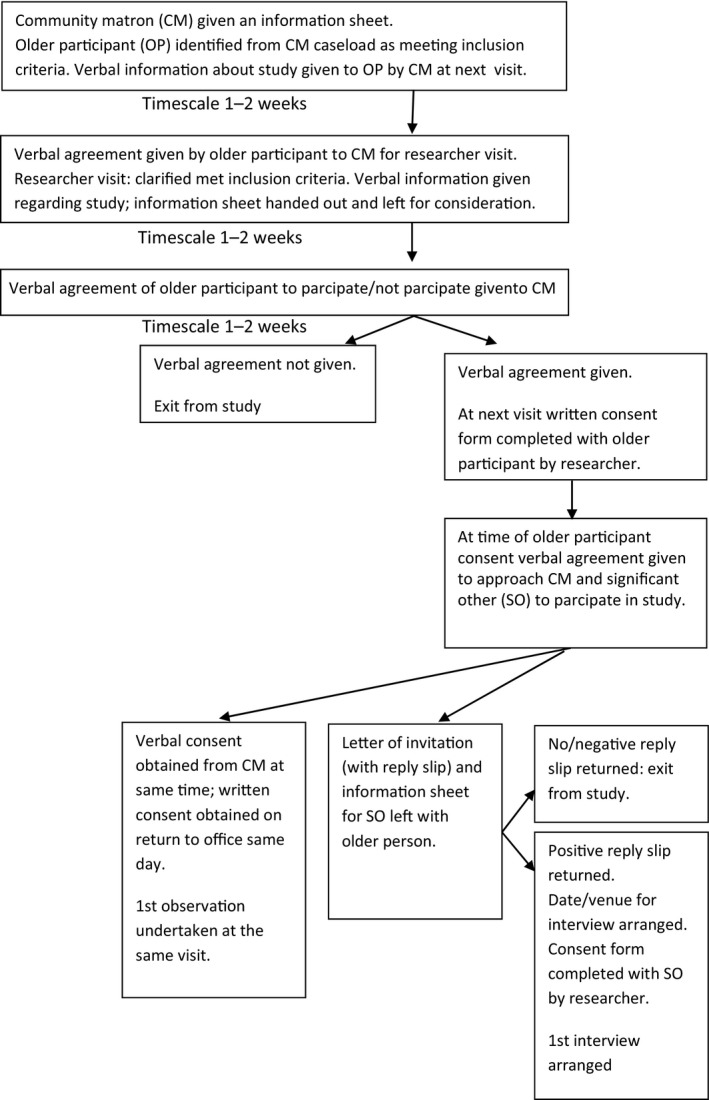
Recruitment of Sample

A purposive sampling technique (Bryman, 2012) was used to identify frail older people who met the inclusion criteria (Box 1). During a community matron visit, an older person was asked whether they were interested in participating in the study. If they agreed, they were provided with an information sheet and asked if a researcher could accompany the community matron at the next home visit for recruitment purposes (Figure 1).

Box 1 **Recruitment criteria**

1 To be eligible for the study, older participants had to meet all the following inclusion criteria:

- Aged 75 and over
- Living at home alone
- Two or more long‐term conditions
- Polypharmacy
- Risk of falls
- In receipt of or eligible for social care services
- Receiving a community matron service
- Perceived as frail by the CM team
- English speaking

### 2.3 Data collection

Participant observation in participants’ homes was conducted by JKS during and following community matron visits. As observer‐as‐participant (Gold, 1958), JKS placed herself in a corner of the older participant's room, observing and recording in field notes the interactions and activities that took place; no care work was involved. Verbal interaction during each care visit was audio‐recorded and written field notes captured what was happening around the verbal interaction (Atkinson & Coffey, 2011). Data collection was completed in December 2012.

Up to six care encounters with each older participant, and their community matron, were observed at monthly intervals, over a period of time ranging from 4 to 11 months. These time frames were sufficient to observe trajectories and facilitated making links between earlier and later time points and contexts (Jamieson & Victor, 2002). For some older participants, the timing between the observations exceeded a month, due to transfers in and out of other care services or a participant becoming unwell.

### 2.4 Semi‐structured interviews

Following the observations, normally a week later, JKS conducted semi‐structured interviews with participants in their own homes. Interview questions derived from the episodes of observation and existing knowledge of frailty were organised into a semi‐structured interview guide (Box 2). Interviews aimed to understand the individual's perspective, linked to the observational data, and to gain insights into the experience of frailty.

Box 2 **Semi‐structured interview guide**

1

1. General introduction to the interview:
  How have you been since I saw you last week?
2. Insight into their daily experiences:
  Could you describe what you do in a typical day?
  What makes daily life difficult for you at this moment and why?
  What impact do these difficulties have on your daily life?
  What makes daily life a positive experience for you at the moment and why?
  How do you try to resolve the difficulties or maintain the positive times?
3. Family/Health and social care support:
  Who is involved in helping you with aspects of your daily life and why?
  How did these agencies/individuals become involved in your daily lives?
  How does the involvement impact on your daily life?
  How do you feel about the problem/help with the problem?
  What are other people's attitudes towards your problems?
4. Understandings of frailty:
  What do you understand by the term “frail”?
  If you had to describe an older person as frail what would that description include?
  In your opinion what has influenced your description of frailty?
  In considering this description would you consider yourself to be frail?
  As the study progressed the interview themes were developed according to data collected during periods of observation and field notes.

### 2.5 Data analysis

Interviews and recorded observations were transcribed verbatim. In order to make sense of the diverse sources of data, we gave equal weighting to each source, for example, the audio‐recordings were listened to alongside reading of the transcript, with subsequent matching to written field notes. This ensured that verbal interactions were considered within context, facilitating a detailed exploration of factors that contributed to frailty (Casey, 2007). Early on in the analysis, we labelled and coded data in an iterative process whereby patterns and sequences of content and experiences over time were identified within and across all the participants. Emergent themes were further developed and refined by analysing similarities and divergences between and within participants (Sangster‐Gormley, 2013).

### 2.6 Rigour

Credibility, dependability and transferability were considered (Holloway & Wheeler, 2010; Polit & Tatano Beck, 2010) using the COREQ checklist (Tong, Sainsbury, & Craig, 2007). Credibility was demonstrated through prolonged involvement and persistent observation in the field (Holloway & Wheeler, 2010). Dependability was achieved by detailing the research process, leaving an audit trail of methodological and interpretative decisions made at each stage of the research process (Elliot, Ryan, & Hollway, 2012). Transferability was enhanced by describing the original context of the research and providing detail of the process and nature of data collected.

### 2.7 Ethical issues

The manager of the community nursing service provided consent to access patients and nurses within the service. Frail older participants and community matrons provided written consent at the start of the study and verbal consent throughout their continued participation. Permission to conduct the research was provided by the NHS Research Ethics Committee and the Local Research Governance Consortium (07/H1308/162). In attempting to recruit frail older people, it was recognised that some potential participants would be vulnerable and that they could feel obliged to take part when asked by the community matron (Pleschberger, Seymour, & Payne, 2011). The initial approach to the older participants was carefully considered (see Box 3 detailing how this was managed).

Box 3 **Access to older participants**

1 In the context of recruitment of older people to the study I was aware that the community matrons were acting as gatekeepers. Therefore, due to the potential power dynamic between the older person and the community matrons, I acknowledged that it might be difficult for an older person to refuse to meet me. This became more of an issue when I realised early on in the field that some community matrons had introduced me as a colleague and not as a researcher. Reflecting upon this, I realised that I did not know how the community matrons actually described me to the potential participants or chose to deliver the information to the older person. Therefore, this may have influenced the decision that an older person made to permit me entry to their home. I picked this up following a visit with the community matron to the fourth potential older participant and wrote in my field notes:

After climbing the stairs we entered the living room, older participant four was sitting in a chair next to the door. The community matron greeted her and went onto mention that I was accompanying her, and as she was sitting down on the settee she went onto say, ‘Do you remember (name of older participant), the colleague that I mentioned last week’. Older participant four nodded her agreement.

Note to self: The CM introduced me as a colleague; I wonder how many other CMs have done this? Must get across my role as researcher.

Following this experience, I ensured that I gave all the community matrons a small card, with details about myself and the study on it. This ensured that all the older people knew that I was a researcher and were given the same information about the study.

## 3 FINDINGS

Ten older people took part in this study. Their age ranged from 77 to 91 years, with a median age of 84 years; seven were women and three were men. All lived alone at home and received care from the community matron service. Table 1 details the study participants, each given pseudonyms to maintain confidentiality.

**Table 1:** Details of the participants including the number of observations and interviews undertaken and the time frame in study

Participants (pseudonym)	Number of observational visits	Hours of observation during CM visit	Number of interviews with older person	Status at end of study period	Time in study (months)
Vera	6	6	6	Deceased	10
Keith	6	8.5	6	Alive	10
Esther	6	7	6	Deceased	11
Grace	3	4	1	Deceased	4
Martha	6	5	6	Alive	9
Derek	6	5.5	6	Deceased	8
Amelia	5	3	5	Deceased	6
Christine	6	7	6	Alive	7
Stephen	6	8.5	6	Deceased	8
Eve	6	4.5	6	Alive	6
Total	56	59	54		

The experience of frailty was understood through the construction of four themes. *Fluctuating ill‐health and the disruption of daily living* details the nature and patterns of transition experienced by the older people in the study. It describes how these transitions contribute to disruption to daily living and the impact they have on the daily lives of older people. *Change in the management of daily living* demonstrates how the older people made adjustments so that they could continue to manage daily living. Taking control of difficult situations was challenging but there was an emphasis on keeping going. *Frailty as anxiety, fear and uncertainty* reveals situations where older people find it difficult to live with fluctuating ill‐health. Accounts of feeling frail illustrate how episodes of uncertainty challenge identity. *Making sense of changes to daily living* explains how older people attempt to come to terms with their current situation, providing insight into how the future lives of older people are shaped by their experience of frailty and ageing.

### 3.1 Fluctuating ill‐health and the disruption of daily living

Many participants experienced physical impairment and incapacity as their overall health declined. The impact of this could be seen in disruption to daily life, which participants generally articulated as loss. For many, this was experienced as not being able to go out and maintain social relationships with family and friends. For others, particularly the women, it was associated with not being able to manage the home environment.

Where older people experienced low mood this appeared to intensify the effects of physical impairment. Over a few months, both Vera and Keith experienced episodes of low mood, and occasionally, this compromised their ability to manage daily living and further disrupted daily routines. For example, as Keith's mood deteriorated, he began to have problems managing his diet and this impacted upon the control of his diabetes.

#### 3.1.1 Ongoing disruption

The majority of participants experienced changes to their health as a constant process characterised by a gradual decrease in their ability to manage daily living and routines over a period of time. Over 4 months, Stephen became increasingly breathless:

On first visiting his home Stephen would open the door when we arrived. A couple of months later he was unable to do this as he became too breathless to walk the distance (4th Observation: Field note extract)

Stephen noticed that his breathing was worsening; 5 months later he described how every day was becoming a struggle:

I felt terrible last night really breathless. I think I had a trying day. Well first thing in the morning I was having a wash and a shave, then I was caught short and I had to dash in there (toilet) with the frame. It went all over the seat….that knocked me back trying to clean that. When I went to bed I felt terrible. In the morning I am breathless, first thing. I can feel myself getting worse (5th interview)

Some participants experienced significant health crises, such as falls or chest infections, and here further disruption to daily routines was encountered. On first meeting Vera she was experiencing a number of health problems, making her increasingly tired, and her mobility was worsening because of her swollen legs:

I'm worried about my swollen legs and feet because they're beginning to affect how I get up and down stairs. I like to rise early in the morning and go downstairs but lately I have had to sit on the edge of the bed and wait for the carers to help me because I've not been able to get down the stairs myself (1st interview)

Over 3 months, Vera's health continued to deteriorate, evidenced by low blood pressure, a number of falls, arthritic pain, constipation and a urine infection, which further exacerbated her decreased appetite and poor mobility. She became increasingly unsteady on her feet, losing her confidence as she was less able to manage her daily living:

I try and make myself a cup of tea, if I can walk in the kitchen……..but this morning I couldn't manage to go in the kitchen as me feet were right swollen. And you know when I'm walking I count a lot, to keep me going, I thought if I count I might keep going me‐self, but I couldn't this morning I was just too tired after getting downstairs (3rd interview)

#### 3.1.2 Stability and disruption

A few older participants experienced a period of stability in relation to their health and well‐being. Although these older participants experienced health problems, they appeared to manage them with no real alteration to daily routines. Derek experienced a chest infection and hypertension during the study period yet he was able to manage these, even when the introduction of a water tablet meant that he had to frequently go to the toilet. Similarly, Eve had frequent episodes of nausea which she managed through decreasing her intake of fatty food and altering the dose of her pain killers for arthritis.

### 3.2 Changes to the management of daily living

To manage the effects of physical ill‐health and impairment, maintaining a daily routine was important. Engaging with such routines created a pattern to the day and all participants attempted to adhere to this. Stephen routinely had an early lunch and then retired to his bedroom to lie down and watch television, conserving his energy for his bedtime routine. Esther would lie down after breakfast to read and after lunch would watch television programmes up to bedtime. Upholding such routines appeared to be significant as it enabled the older participant to sustain their sense of self as an independent person and a degree of continuity with important aspects of life was maintained.

All had developed coping strategies to deal with the episodes of fluctuating ill‐health, as well as being supported in varying degrees by informal and formal social support networks. Regardless of the extent of ill‐health and disability the older participants strived to maintain their daily routine. This involved managing new episodes of, or worsening, ill‐health and the subsequent challenges that these brought to maintaining independence. Ongoing and increasing impairments and constraints were rethought in order to accomplish daily routines. To manage her decreasing mobility, Vera began to eat tray meals on her lap rather than walk to the kitchen table.

Taking additional time to complete tasks was another approach used. As Esther's breathlessness became worse, she found it harder to walk to the kitchen from her downstairs bedroom. She continued walking to the kitchen but rested for longer periods so that she could get her breath back, as well as changing activities:

I no longer make a cup of tea; I only take my pots through on the trolley for the carer to wash. They make me a flask of tea at lunchtime so that I can drink that for the rest of the day (4th interview)

Omitting daily tasks altogether was the only strategy that some participants could employ to manage their symptoms. This was the case for Amelia, who during the last 3 months of her life was consistently unable to have a daily bath or wash. She did not persist in attempting this which enabled her to have the energy to interact with her daughter and grandchildren when they visited.

#### 3.2.1 Keeping going

Despite the decline in health, the older participants continued to attempt activities that contributed to their daily routines. Efforts to take control were tempered by being realistic about what they could or could not do, in any given situation. Disengaging completely from these activities did not appear to be an option. Adopting a positive approach appeared to enable the older person to “keep going,” and small accomplishments were viewed as important to continue with daily routines. A comment by Christine during her transition in health summed up the positivity and resilience many older participants exhibited:

If I can carry on and cope one way or another I will (5th interview)

### 3.3 Frailty as fear, anxiety and uncertainty

On occasion, the effort required to manage daily routines pushed the capabilities of some older participants to the limit, particularly in a crisis. Although circumstances where participants found it challenging to manage daily living were unique to each individual, a feature of these situations was that they provoked anxiety and fear; some articulated that they felt frail.

Where bodily changes made it difficult to negotiate daily living, previously taken‐for‐granted activities were perceived as risky. Some participants became apprehensive about being able to go about their daily business. Following Martha's fall, resulting in a broken leg, she became frightened of falling again and sustaining further injury, mentioning 2 months later:

I do feel frail sometimes. There have been times when I have been walking with my stick and I've felt as though I'm going to trip you know. I'm frightened of tripping. I've always been a dare devil even as a child. But it has bigger consequences; well I found out that myself (3rd interview)

Despite feeling afraid, Martha did not stop taking risks with her mobility during her rehabilitation. During her fourth interview, she recalled how she was waiting for the better weather so that she could walk in the garden alone. Her attitude to risk taking appeared to change as she regained control of her body.

Similarly, Grace found it increasingly difficult to accommodate the changes to her body following a series of falls. On return home from an intermediate care unit admission, Grace worried about how she was going to physically manoeuvre around her home. She described how she had been unable to get up from the toilet:

G I've had a high seat toilet put in today; I couldn't get off the toilet yesterday when I came home. And I thought, “Oh lord.” Anyway I sat and sat. I'd been sat there an hour and I couldn't get off and I thought, “Oh don't panic someone will come this afternoon.”

R And you were sat on the toilet for 1 hr?

G Yes.

R Oh Grace.

G That's what I thought, but I just couldn't move you see to get out of the way. I tried and tried but then gave it up as a bad job

(2nd interview)

For some, situations that signalled that their health was worsening caused anxiety and distress. Christine described her experience of an unexpected episode of severe chest pain:

I had a really bad angina attack last week. It woke me up in the night; the pain woke me up. I thought, ‘What's wrong with me at this time in the morning?’ I was really scared. I think I panicked because I didn't know what was happening. And I'm worried now what it means (3rd interview)

Uncertainty linked to changes in a participant's health condition and ability to manage daily routine was expressed in other ways. Vera became tearful during observations towards the end of the study as she was increasingly overwhelmed and distressed by the changes to her health. She often mentioned that she did not know what was wrong with her, “*I don't know why I am like this.”* Latterly, Esther's conversations were dominated by frustration, and occasionally anger, at her increasing breathlessness as it indicated to her that her condition was getting worse. Grace became agitated by the deterioration in her health during later visits and she constantly repeated the phrases, “*when I adjust”* or “*when I adjust I will be alright.”*

Where the participants were aware of changes to their bodily health and the risks such changes imposed, they appeared vulnerable as their sense of self was threatened. For some older people, episodes of feeling frail were accommodated and therefore became a temporary feature of their experience. For others, the situations became more permanent.

### 3.4 Making sense of changes to health and daily living

Reappraisal of ongoing changes to health and disruption to personal routines, in relation to an ageing self, enabled the older participants to further redefine their problems and accommodate changes to daily routines. Esther constantly described herself as being “*past my sell by date,”* and how she had come to terms with not being able to go out by lowering her expectations in the light of her advancing age:

I'm not worried about all that now. I'm quite content to sit here and watch the world go by, watch my telly and read, that's all really. I've accepted that I'm 88 and a half. They can't do anything about that, nobody can. So I shake myself, I think pull myself together. There's nothing I can do about my age. So I've accepted it all (4th interview)

Using age as a barometer and making comparisons with older people who were less able was a common feature of the data, particularly regarding mental capacity. When contemplating his deteriorating health, Stephen considered that he had had a *“good innings”* and he was better off than many others as he had retained his *“faculties.”* These comparisons seemed to be used as a benchmark for being able to positively reshape ideas of independence in the face of disruption.

Awareness of becoming “old” was a complex process and not always readily accepted, compounded when the experience of transition appeared to be sudden and chaotic. As Martha's recovery from a fractured femur continued, it became evident she would be left with a degree of permanent disability. Martha had never considered herself old and resisted the offer of a stick as it represented getting older:

I didn’t want a stick, because old people have sticks I don’t feel any different in myself, I don’t feel old(6th interview)

For some, the experience of bodily decline and deteriorating health led them to contemplate the end of their lives. Stephen, Amelia and Martha talked openly about death. Stephen and Amelia both expressed the desire for their lives to end. Over 2 months, Amelia experienced a number of near death experiences:

I had me tablets and things. I went and had me nebuliser. I walked into the kitchen, I usually count, something to take me mind off it, I can't remember if I got to 25 or 50. I couldn't breathe so I went back into there on the machine. I started panicking couldn't breathe and [care worker] came in, I must have been drifting in and out of consciousness. Depending which way you look at it, it isn't my time to go, but I wish it was. I'm weary (3rd interview)

In contrast, although Martha began to consider her own mortality, she was not ready to die. She had contemplated death when she had first suffered two heart attacks, but during her recovery, the motivation to see the birth of her latest great grandchild kept her going.

## 4 DISCUSSION

The findings of this study indicate that frail older people experience transitions in health and illness as a continual process of change, with sudden events, general decline and periods of relative stability frequently intersecting. Existing perspectives on transitions in health and illness in later life often portray them as single, phased and linear events (Torres and Hammarstrom et al. 2006; Godfrey & Townsend, 2008). In contrast, this study suggests that frail older people's experiences of transition are neither single event nor do they neatly fit into a given linear category at any one time, also identified by Grenier (2012).

The findings presented here show how the perpetual state of transition endured by older people influences the ways in which they manage and adjust daily living routines. This resonates with research by Grenier (2012) who identifies how frail older people seek to achieve a balance between their disability and impairment and ability to manage daily routines. Achieving a balance for the older people in this study involved them modifying existing patterns of daily living and creating new ways of managing. This occurred even when transitions were enduring and multifaceted in nature, often pushing an older person's adaptation to the limit. These findings build upon theoretical perspectives illuminating older people's capability to work with significant disruptions to daily living and accumulated loss (Hicks, Sims‐Gould, Byrne, Khan, & Stolee, 2012; Janssen, Regenmortel, & Abma, 2012; Lloyd, Calnan, Cameron, Seymour, & Smith, 2012; Nicholson et al., 2012).

In striving to construct and conserve daily routines, the older people in this study did not live up to the stereotyping associated with frailty (Nicholson, Gordon, & Tinker, 2017). Sustaining daily routines and renegotiating activities and priorities contributed to the maintenance of an older person's identity as an independent person. This finding concurs with research on the exercise of personal agency by frail older people (Grenier 2007, Hammarstrom & Torres, 2010; Breitholtz, Snellman, & Fagerberg, 2013; Warmoth et al. 2016) and on resistance to objectification and stereotyping associated with ageing and frailty (Nicholson et al., 2012; Van Campen, 2011). Making choices in day‐to‐day living enabled these older people to continue to engage with life, providing a counterpoint to the idea that the visibility of agency decreases, as older people enter the fourth age (Higgs & Rees‐Jones, 2009).

As found in other studies (Lloyd et al., 2012; Portacolone, 2013), as daily living became precarious, the participants experienced episodic moments of fear, anxiety and uncertainty, sometimes feeling frail. The balance was generally tipped by bodily decline, echoing other research (Grenier, 2007) and changes to mental health and well‐being further exacerbated physical decline (Hurd Clarke, 2010). Older people's fear and anxiety also linked to being alone; illustrating the multifactorial nature of frailty (Nicholson et al., 2012). Our findings suggest that older people anticipate a situation where independence may decrease and the ability to exercise personal agency is reduced, similar to Grenier's (2006, p1) description of “rupture of self.” In this study, “rupture of self” led older people to contemplate their ability to continue living at home, their own ageing and mortality.

This study illuminates the disconnection between frail older people's experiences and current health policy. Case finding, assessment and individualised care and support plans, underpinned by the principles of Comprehensive Geriatric Assessment (CGA) (British Geriatric Society (BGS) 2014), are the cornerstone of policy shaping the care of frail older people living at home (BGS 2014; NHSE 2017). These policies emphasise using a suite of tools, including a brief CGA, to assist with case finding, assessment and subsequent support, including annual review (Clegg et al., 2016; NHSE 2017). Yet the overarching focus on impairment, problems and dependency within these frailty tools is at odds with the older person's experience in this study. Older people were aware of what they could not do for themselves and how this fluctuated. However, they focused on continuing to engage with daily living and in doing so they defined themselves in relation to what they could achieve, as observed by Nicholson, Gordon et al. (2017). It is imperative therefore that frailty tools capture the positive and diverse ways in which older people manage their daily lives to optimise integrated care and support. Otherwise assumptions about what people can or cannot do based on functional impairments will continue to drive service provision.

These findings identify that frail older people do experience anxiety and fear and may feel frail, when daily living becomes challenging. In the context of a personalised approach to assessment and care planning (Coulter, Roberts, & Dixon, 2013; NHSE 2014), there is therapeutic potential in exploring the emotional experiences linked to a frail older person's interpretation of events when they experience anxiety and fear, or appear to be living with uncertainty (Grenier, 2006). Here, specific examples of living with frailty, particularly during transitions in health and illness, can focus and maximise opportunities for providing integrated care that supports a frail older person's vulnerabilities (Age UK & British Geriatric Society, 2015). Increasingly the role of the Advanced Nurse Practitioner (Goldberg, Cooper, & Russell, 2014), including community matrons, is being mooted as one way in which the complex needs of frail older people living at home can be met. The advanced interpersonal and clinical assessment skills that community matrons utilise (Savage, 2012) could be integral to identifying how older people live with frailty, creating and sustaining a person‐centred approach to care within the integrated team.

## 5 STRENGTHS AND LIMITATIONS OF THE STUDY

This study enabled older people, generally confined to home, to express their views and opinions about living with ill‐health, impairment and frailty. Therefore, the findings offer insights into the experiences of some older people for health and social care practice and policy development. However, what is missing is the voice of the older person who is unable to communicate easily, for example, those living with dementia or stroke. Although some of the findings from the study are likely to be intensified in the presence of such conditions, we do not have the data to be sure. The community matrons subjectively determined the presence of frailty; an objective measurement would have enhanced the recruitment of frail older people. The position of researcher and the influence this had on the study's development and process is acknowledged. The first author is a nurse educator, with a focus on teaching ageing and palliative and end‐of‐life care. Through reflexivity, the complexities of the researcher presence, and the influence on the process of data collection and interpretation of the findings were considered.

## 6 CONCLUSION

This study has contributed to the understanding of older people's personal experience of frailty in later life. Older people work to shape and maintain daily routines in the context of complicated and enduring transitions in health and illness. However, older people experience episodic moments of frailty in their daily lives, often articulated as uncertainty, where daily living becomes precarious and resilience is threatened. Developing an understanding of the personal experiences of frail older people in the context of transition has the potential to inform nursing practice in person‐centred care. These findings have implications for policymakers and those who deliver care to frail older people.

## CONFLICT OF INTEREST

There are no potential conflicts of interest.

## CONTRIBUTIONS

JKS, JS, TA contributed to the overall design of the study, data analysis and preparation of the manuscript.
